# Mobilizing for Health: A Case Study of Kazakhstan’s Vaping Ban Advocacy Campaign

**DOI:** 10.3390/ijerph22071102

**Published:** 2025-07-13

**Authors:** Jamilya Sadykova, Akerke Ayaganova, Kuanysh A. Yergaliyev

**Affiliations:** 1 National Coalition ‘For Smokefree Kazakstan’, 010000 Astana, Kazakhstan; dzhamilya75@mail.ru; 2 Strategic Planning Department, Aktobe Regional State University Named After K.Zhubanov, 030000 Aktobe, Kazakhstan; akerke.ayaganova@zhubanov.edu.kz; 3 Department of Medicine, School of Medicine, Nazarbayev University, 010000 Astana, Kazakhstan; 4 SDU Business School, SDU University, 040900 Almaty, Kazakhstan

**Keywords:** public health advocacy, adolescent health, vaping ban, power prism framework, Kazakhstan, tobacco control

## Abstract

This article focuses on an advocacy campaign for a complete ban on vaping in Kazakhstan led by the Smokefree Kazakhstan Coalition. Initiated in 2021, the campaign aimed to address the growing public health concerns about vaping among adolescents, which was reflected in a sharp increase in vape use among young people—from 1.6% in 2014 to 5.8% in 2022. Despite facing strong opposition from the vaping industry and political lobbyists, the Coalition gained support from key political figures, public health leaders, and NGOs. Over 32 months, the campaign achieved several key milestones, including the inclusion of criminal liabilities for those involved in the vaping industry, and, in 2024, it eventually joined a number of countries such as Thailand, Qatar, Japan, Singapore, and India in approving a vaping ban. The advocacy efforts relied on public engagement, social media, and coordinated civil society mobilization—including petitions, public meetings, awareness campaigns, and coalition-building among NGOs and health advocacy groups—to overcome industry resistance toward official vaping market ban approvals. This article uses the case study approach with the Power Prism framework to describe and evaluate the advocacy campaign’s strategic plan, its political challenges, and the significant impact of public health advocacy in shaping national health policy. The significance of the article lies in the success of the vaping ban in the Kazakhstani context, which may serve as a model for other countries facing similar public health issues, political instability, and industry resistance.

## 1. Introduction

### Vaping Crisis in Kazakhstan

The growing use of electronic cigarettes (commonly referred to as e-cigarettes, e-cigs, or vaping products) has become widely acknowledged as a global public health concern [1,2]. Kazakhstan is also facing a growing public health crisis with the rise of vaping, especially among its young people. Once perceived as a safer alternative to traditional smoking, vaping has rapidly become an epidemic among children and adolescents globally, including in Kazakhstan [3].

International and national studies on smoking trends among the population, such as the Global Youth Tobacco Survey (GYTS) 2014 and Global Adult Tobacco Survey (GATS) 2019, the National Report on Health Behavior in School-aged Children (HSBC) 2018, National Data 2023, and research conducted by scholars, provide valuable insights into the smoking habits of Kazakhstani adolescents, with a focus on various tobacco products and electronic cigarettes [4,5,6,7,8]. According to these studies, the proportion of adolescents in the country who use vapes has nearly tripled between 2014 and 2023 [4,5,6,7,8].

**Figure 1:**
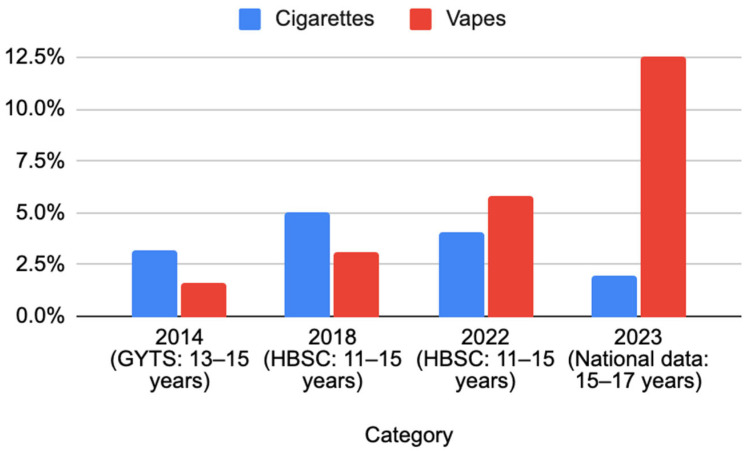
The outcomes of international and national studies on smoking rate among adolescents in Kazakhstan between 2014 and 2023.

As illustrated in Figure 1, in 2014, according to the GYTS, 3.2% of adolescents aged 13–15 were current cigarette users, while only 1.6% used vapes [9]. It is important to note that the data presented in Figure 1 are drawn from different studies, which limits the direct comparability of these figures. According to the GYTS Indicator Definitions, “current use” refers to the number of respondents who had smoked cigarettes or any other smoked tobacco products in the past 30 days [10]. By 2018, data from the HBSC study showed an increase in both categories among 11 to 15-year-olds, with 5% smoking cigarettes and 3.1% using vapes. However, in 2022, the trend began to shift. While cigarette use slightly declined to 4.1%, vape use saw a sharp increase to 5.8%. By 2023, national data for older adolescents (15–17 years) revealed a dramatic change. Regular cigarette use had dropped to 2%, marking the lowest rate in the observed period. In contrast, vape usage had surged to 12.5%, indicating a growing preference for e-cigarettes over traditional tobacco.

Overall, while cigarette smoking has generally declined among adolescents in Kazakhstan, current vape use has escalated, particularly among older adolescents. This shift reflects global patterns, where e-cigarettes are increasingly popular among young people due to perceptions of reduced harm, attractive flavors, and marketing strategies. The sharp rise in current vape use calls for targeted public health interventions to address potential risks and prevent nicotine addiction in young populations.

**Figure 2:**
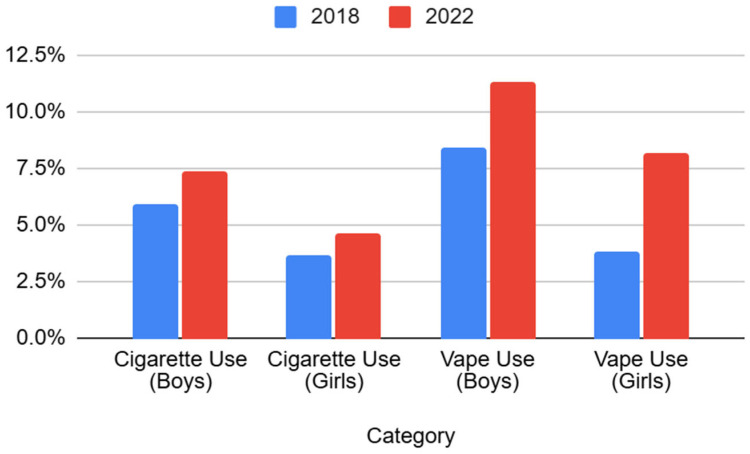
Increase in cigarette and vape experience between 2018 and 2022.

Figure 2 shows that, according to the HBSC 2018 and 2022 reports, between 2018 and 2022, both current cigarette and vape use among boys and girls tended to increase in Kazakhstan, but the magnitude of increase was significantly greater for vaping [11,12]. Cigarette smoking experience among boys increased by 1.5% (from 5.9% to 7.4%), and it increased by 0.9% for girls (from 3.7% to 4.6%). In contrast, vaping experience rose by 2.9% among boys (from 8.4% to 11.3%), whereas the increase among girls was more pronounced, more than doubling from 3.8% to 8.2%. These trends highlight a sharper rise in vaping than in cigarette smoking, especially among girls, indicating a shift in nicotine consumption preferences among adolescents.

In Kazakhstan, there is a clear trend of decreasing numbers of children and adolescents smoking regular cigarettes, accompanied by a concerning and rapid increase in the number of children using vapes (electronic cigarettes). Compared to Global Youth Tobacco Survey data, the number of cigarette smokers among adolescents aged 13–15 in Kazakhstan increased from 2% to 5% among boys and from 1.3% to 2% among girls. The number of e-cigarette users increased threefold among boys—from 2% in 2014 to 6% in 2018—and twofold among girls—from 1.1% to 2%.

The widespread availability of marketing for flavored electronic cigarettes has fueled an epidemic of vaping, which has also led to a troubling trend of dual consumption—both cigarettes and vapes—among adolescents. Several studies have indicated that this trend is contributing to an increase in cigarette smoking among young people [13,14,15]. These developments highlight the urgent need for timely and comprehensive measures to curb the growing epidemic among adolescents, addressing the widespread use and accessibility of electronic cigarettes.

Growing concerns over this trend mobilized public health advocates in Kazakhstan to push for stronger regulations. Since October 2021, the Smokefree Kazakhstan Coalition (the Coalition) had been spearheading an advocacy campaign aimed at achieving a complete ban on vaping products, including flavorings and liquids. This effort is rooted in data showing the toxic effects of vaping and its increasing popularity among school-aged children. The campaign not only focused on public health concerns but also worked to combat the influence of the vaping industry and its lobbying efforts.

Therefore, the purpose of this article is to describe the advocacy campaign that led to the complete ban of vapes in Kazakhstan, and to evaluate its design, strategies, and outcomes using the Power Prism Advocacy framework.

## 2. Materials and Methods

### Conceptual Framework

**Figure 3:**
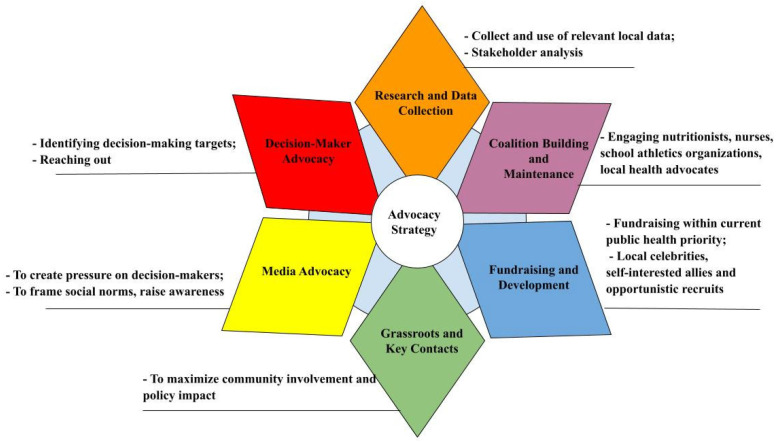
Power Prism framework. Adapted from Yergaliyev, K. A.; Fresina, L.; Ayaganova, A.; Austin, S. B. Power Prism Framework for Health Advocacy: A Case of Dietary Supplements for Weight Loss and Muscle Building Sold to Youth in Massachusetts Municipalities. Electron. J. Gen. Med. 2025, 22 (4), em662. https://doi.org/10.29333/ejgm/16371 [16].

This study employed a case study approach to examine the advocacy campaign for a comprehensive vaping ban in Kazakhstan. The primary sources of data included official government reports, parliamentary meetings, public petitions, media articles, personal experience of coalition members and coalition meeting minutes, and relevant research studies. These data were analyzed, synthesized, and organized using the Power Prism framework (see Figure 3). The Power Prism framework was chosen for its structured approach and unique ability to organize and explain the advocacy campaign clearly [16]. Although the framework itself was not directly applied during the campaign implementation that led to the vaping ban in Kazakhstan in 2024, its step-by-step structure and six “power tools” offer a valuable lens that allows us to analyze, understand, and communicate the campaign’s strategy and outcomes effectively.

As a first step, the framework proposes considering the following three key questions for advocacy campaigns: (1) what do you want, (2) why do you want it, and (3) who has the power to give it to you? In answering these questions, the campaign primarily aimed to achieve a full vaping ban in Kazakhstan, including criminal liability for the import and sale of vapes. Secondly, the campaign advocated for this ban due to significant emerging health issues among young people and the rapid rise of a vaping epidemic among them. Thirdly, key political figures at the government and parliamentary levels, along with the Minister of Health (MoH), served as the primary decision-makers who had the authority to implement the full vaping ban.

The framework also facilitates thinking about advocacy activities and events through the following “power tools” to build more power for the advocacy mission [16]:

- Research and data collection tools require the collection of all relevant information that addresses the problem and reveals its distinctive features.
- The coalition-building and maintenance tool proposes the formation of a coalition of diverse individuals and organizations to support advocacy efforts, thereby increasing the likelihood of a campaign’s success.
- Funding and development tools urge reflection on the financial resources needed to organize advocacy events.
- Grassroots advocates and key contacts are important individuals, as they are constituents of elected officials or may have access to key decision-makers.
- Media advocacy tools should be utilized to engage key decision-makers and the general public.
- A decision-maker advocacy tool involves advocates identifying the individuals responsible for making necessary decisions and attempting to influence them through traditional lobbying methods.

In contrast to other advocacy frameworks, the Power Prism framework considers these six strategic power tools to be necessarily interconnected and focuses on specific political landscapes and community needs.

## 3. Results

### 3.1. Research and Data Collection

To build a strong advocacy campaign, the Coalition members collected all relevant data from national and international reports such as the GYTS, HSBC, and National Data available from 2014. Evidence-based international data, such as a 2021 study by researchers at Johns Hopkins University revealing that vaping aerosols contain nearly 2000 distinct chemicals, as well as local data described below, became a central message of the advocacy campaign [17]. It was crucial to emphasize the link between vaping and serious health conditions, such as EVALI, a severe respiratory illness responsible for multiple deaths globally [17]. Despite the fact that not all vapes contain Vitamin E acetate (VEA), it has been commonly found in illicit THC vape products and is strongly linked to EVALI due to its toxic effects when inhaled [18,19,20]. Wu & O’Shea et al. [20] also demonstrated that heating VEA in vape aerosols produces the toxic gas ketene, which can damage the lungs, while a 2020 MDPI review and a report of Centers for Disease Control and Prevention (CDC) describe how VEA and vaping oils disrupt lung surfactants [18,21,22].

The most alarming aspect of vaping in Kazakhstan was its rapid adoption among young people. National surveys, such as the HSBC research, revealed a sevenfold increase in vaping among schoolchildren aged 13–15 years since 2021 [11]. The Coalition capitalized on these data, framing vaping as a public health crisis that disproportionately affected Kazakhstan’s future generations. Like earlier anti-tobacco campaigns driven by a coalition focusing on youth smoking, it made protecting children and adolescents a central argument in advocating for a complete ban [23,24]. The Coalition’s efforts highlighted how the vaping industry deliberately targets young people with flavored products and sleek, technology-driven designs and broad accessibility of toxic products.

A core theme of the campaign was the protection of young people, with advocates highlighting the rise in vaping among minors as a critical reason for implementing the ban. A central goal of the campaign’s youth protection narrative was the prevention of nicotine addiction and related long-term health consequences among adolescents. Advocates emphasized that banning flavored e-cigarettes and vape products would significantly reduce their territorial and financial accessibility, particularly for minors, thus lowering usage rates. The campaign stressed that early initiation into vaping increases the risk of lifelong nicotine dependence, which is associated with respiratory illnesses (including EVALI), cardiovascular disease, and other non-communicable conditions [25,26]. Additionally, it aimed to address poly-use behavior among young people—simultaneous use of e-cigarettes, traditional cigarettes, and heated tobacco products—which compounds health risks. The protection argument also resonated with societal concerns, as increasing numbers of parents reported frustration over the state’s perceived inaction in regulating vape products. Finally, advocates pointed to the environmental harms of vapes—such as plastic and battery waste and aerosol emissions—further framing the ban as a safeguard for both youth health and the broader ecosystem.

Another valuable source of information utilized by the Coalition was market-related data highlighting the widespread availability of vapes nationwide, driven by their low cost and extensive distribution [27]. It is worth highlighting that accessing the market-related data was challenging due to the lack of cooperation from the relevant authorities. However, the Coalition obtained detailed import data from the Kazakhstani Ministry of Finance in a raw Excel format, without any statistical processing. Coalition members manually calculated and analyzed the figures for inclusion into the regulatory risk assessment (RRA/RRAs), and the analysis revealed that the ESP/vape market grew by nearly 300% between 2020 and early 2022 [28]. The RRA is legislation expertise that examines how the introduction of new regulations may impact entrepreneurs and businesses in the country.

Specifically, official data revealed an extraordinary increase in imported vape units—from just 21,608 in 2020 to 6,301,623 units by early 2022 [28]. Furthermore, the actual size of the shadow market for vape imports could be significantly larger, given the high financial incentive (estimated to be at least USD 6 or more per vape unit) [28]. Even the pro-industry NGO, the League of Consumers of Kazakhstan, acknowledged the rapid market growth and easy accessibility of vapes to children. In the first six months of 2022 alone, vape sales in the country’s two largest cities—Astana and Almaty—increased by 372% [19,28].

In addition to market data, the Coalition actively leveraged findings from a targeted investigation conducted by the MoH regarding the nicotine content in vaping liquids. This investigation revealed alarmingly high nicotine concentrations—up to 67.6 mg/cm^3^ per vape liquid unit, nearly 68 times greater than the permitted nicotine limit for vapes (1 mg/cm^3^) and significantly higher than the nicotine content in an average cigarette (0.7–0.9 mg) according to the MoH Order from 15 December 2020 [27,29].

Furthermore, the Coalition referenced data from the Global Adult Tobacco Survey, highlighting widespread misconceptions about vaping as a safer alternative to cigarettes [5]. According to this study, adults primarily cited pleasant aromas (75.6%), the belief that vaping is less harmful than tobacco smoking (72.7%), and enjoyment (68.3%) as reasons for using vapes. Importantly, 20.4% of smokers reported being unaware of whether vapes even contained nicotine. This clearly indicated a low level of public awareness regarding the composition of and health risks associated with vapes, as well as a general lack of concern about potential health impacts. Thus, local data, along with statistics on e-cigarette smoking among teenagers, became compelling reasons to call politicians to act and make the necessary amendments to the Health Act of Kazakhstan to ban vapes in the country.

### 3.2. Coalition-Building and Maintenance

Each coalition requires a leading organization supported by dedicated staff members and volunteers who implement campaign tasks and monitor their timely completion. In this vape-banning initiative, the leading organization was the “Smokefree Kazakhstan” Coalition, which successfully engaged multiple stakeholders in the advocacy campaign. These stakeholders included government entities (with the MoH playing a central role), non-governmental organizations (NGOs), public health and religious leaders, healthcare professionals, and parents.

The Coalition was established in 2005 by public health leaders (Akanov A, Izmukhambetov I, Sadykova J) and 25 medical, governmental, and non-governmental organizations with the mission to strengthen public health and advance tobacco control by uniting the efforts of civil society around shared interests [30]. The Coalition aims to develop unified strategies and positions on tobacco control at national and regional levels, foster partnerships with government and international organizations, support the implementation of the WHO Framework Convention on Tobacco Control, and promote public awareness, policy development, and coordinated advocacy against the tobacco industry. The professional team of the Coalition has engaged in research, advocacy, and media relations on issues of tobacco consumption reduction, capacity building, and tobacco control policy development.

In 2020, the Coalition successfully advocated for the passage of Article 110 Tobacco Control of the Healthcare Act, which forced the implementation of strict regulations such as a ban on the display of all tobacco products at all points of sale, a complete ban on the smokeless tobacco market, and the treatment of new tobacco products (HTPs, vapes, hookah) as cigarettes with a ban in public places, including outdoor playgrounds and underage driving in the cabin [31]. The coalition also initiated 65% pictorial health warnings for HTPs and hookahs and a 3–4× increase in fines for all tobacco-related violations. This achievement represented a historic breakthrough and marked a significant victory in Kazakhstan’s fight against tobacco—an accomplishment widely recognized by the international community [32].

One of the initial steps taken by advocates to form the anti-vaping coalition was to secure support from diverse societal groups, especially parents and religious organizations such as Muslim and Christian communities. These groups viewed vaping as both a moral issue and a significant social concern and later played a crucial role in public debates. Healthcare professionals were represented by four practicing pulmonologists and five scientists specializing in epidemiology and public health, all of whom played a key role in shaping the political debate at both governmental and parliamentary levels.

The main political ally of the Coalition was the MoH of Kazakhstan, which had a complete vaping ban at every level of political decision-making. Coalition support was reflected in a final official report which summarized research findings, credible industry data, and recommendations. The strong tandem of the Coalition and the MoH directly impacted the success of advocacy efforts throughout the campaign.

Notably, the campaign faced significant political resistance, particularly from the vaping industry and its associated lobbyists. There was a group of notable opponents within the lower chamber (the Majilis) of Kazakhstan, who consistently argued for weak regulation rather than a full ban. Their stance echoes the tactics used by the tobacco industry in the 1990s, which argued that regulation, but not prohibition, would control public health issues related to smoking. These pro-vaping members of parliament have worked to delay the passage of RRA by the government and soften penalties related to the ban.

Overall, the Coalition was structured as a diverse and robust network comprising 20 active stakeholders united to address and end the vaping epidemic: two parents’ NGOs represented by seven highly active parents, five scientists specializing in epidemiology, four practicing pulmonologists, four prominent religious leaders, two lawyers, the dedicated Coalition implementation team, and institutional partners such as the MoH, the Ministry of Internal Affairs, and the World Health Organization. The Coalition implementation team was composed of the leader of the Coalition who was medical doctor by training, one manager who was responsible for managing documentation and coordinating approval processes within the MoH and the government, and a journalist who was a former cardiologist and played a central role in refining texts, drafting press releases, speaking at online events, monitoring media coverage, and promoting the Coalition’s petition.

### 3.3. Fundraising and Development

The primary funding for the campaign was provided by the MoH of Kazakhstan, which helped ensure its independence and credibility. The Coalition routinely received some modest funding from the MoH from 2020 that allowed it to cover the salaries of staff members involved in field inspections. Importantly, financial support was not a central element of the campaign, and advocates deliberately avoided funding sources that could pose conflicts of interest or undermine the campaign’s integrity.

### 3.4. Grassroots Advocates and Key Contacts

When leading an advocacy campaign, it is crucial to employ a bottom-up strategy that emphasizes elevating the voices of local communities. Grassroots advocacy specifically involves coalition members directly engaging and mobilizing individuals who are most affected by the policy issue and who can effectively influence lawmakers. Typical grassroots advocacy tactics include online petitions, patch-through calls, digital advertisements, social media campaigns, and other outreach activities designed to maximize community involvement and policy impact.

Like previous successful tobacco control efforts, this vaping ban campaign heavily relied on active public engagement. The Coalition strategically involved civil society organizations, particularly those representing medical professionals and parents, to ensure that public opinion significantly influenced the campaign. Participation from mothers and health experts during public hearings was especially impactful, as their compelling testimonies publicly exposed and dismantled industry-backed manipulative arguments.

Parents led by Bayan Akhatai, Deputy Chairperson of the Council of Mothers under the Assembly of the People of Kazakhstan, initiated an online petition addressed to the President of Kazakhstan. This petition gained remarkable momentum, gathering over 21,000 authentic signatures, underscoring the campaign’s widespread grassroots support and credibility [33]. It was effectively disseminated among parents through WhatsApp, social media platforms, personal contacts, and public meetings. The petition was authenticated by requiring each supporter to provide their full name, residential address, and most importantly a unique comment explaining their position.

The voices of parents were particularly powerful and effective in raising public awareness about the vaping epidemic among schoolchildren. They emphasized that despite existing legal restrictions, vapes remained easily accessible to children and were frequently marketed in ways specifically designed to appeal to younger audiences. Additionally, their message highlighted the struggle parents and schools faced in addressing the rapidly growing issue of nicotine addiction among children.

In response to parents’ petitions, the vaping industry launched its own counterpetition, claiming to have gathered more than 100,000 signatures. However, upon thorough investigation, it became clear that this petition had been fabricated [34,35]. Analysis of the petitions and accompanying comments revealed that they had been artificially generated, with multiple comments authored by the same individuals and falsified voting figures [34,35]. Coalition representatives publicly exposed this fraudulent initiative in the media, effectively neutralizing the industry’s attempt to undermine the advocacy campaign [36].

### 3.5. Media Advocacy

The role of media advocacy while leading the campaign was crucial since it helped amplify the campaign’s message to reach a much wider audience than direct outreach alone. Through the strategic use of various media channels, health advocates can influence public opinion and generate broader support for their cause. Moreover, media coverage can put pressure on decision-makers and policymakers by bringing issues into the public spotlight. When an issue receives significant media attention, it may compel authorities to respond and take action.

In the case of this vaping ban campaign, the Coalition utilized public hearings to bring together NGOs, health experts, and parents to discuss the risks associated with vaping. These hearings, held both in person with preselected leaders in the medical and parent communities and via online platforms, became essential in amplifying the voices of ordinary citizens, particularly mothers, who were among the strongest proponents of the ban.

The Coalition did not spend any financial resources on media advocacy and made great use of free social media and other available platforms to increase public and political attention. Facebook posts, combined with traditional media reporting, helped portray vaping as a public health issue that requires urgent political resolution through legislative amendments.

Facebook emerged as a crucial platform for the Coalition, allowing it to identify and expose specific individuals who actively supported this harmful industry openly. It was a unique case that highlighted the significance of the vaping issue in the country, allowing the campaign, without spending substantial finance, to attract widespread attention from a diverse audience and media outlets, many of which sought comments from the Coalition on the topic.

In addition to online campaigns, public hearings helped counter industry misinformation and mobilize public support for the ban at the highest level. The Central Communications Service (CCS) under the President of the Republic of Kazakhstan became a strong platform for the Coalition. In May 2023, the Coalition invited various representatives from the MoH, the Mothers’ Council, the National Coalition, senior doctors, and leaders of Muslim and Christian faiths to speak at the CCS. The diverse and unique experiences of the speakers at the CCS media briefings demonstrated the willingness of society to influence the protection of the health of the nation and young people from negative consequences [36].

In October 2023, on the same platform, another influential group of representatives from various fields, such as the Kazakhstan Representative Office of the World Health Organization, the Union of Lawyers, the Police Department, the MoH, the Mothers’ Council, and the National Coalition, discussed loopholes in the law via the example of hookahs, which were manipulated by the vaping industry to overturn amendments to the law [37]. The industry used poor enforcement of the hookah smoking ban in public places to convey the message that market bans in general would not work. However, the Coalition members identified the differences between a full ban and partial ban, the gap between a smoking ban and a market ban, loopholes in the law, and the true reasons for poor enforcement that need to be addressed when implementing a vaping ban. The members also used international best practice in enforcing the smoking ban; therefore, criminal liability was again justified as a tool for strict enforcement.

### 3.6. Decision-Maker Advocacy

Decision-maker advocacy activities were vital for creating meaningful change. Decision-maker advocacy creates direct channels of communication with those who have the real power to implement changes. Regular interaction and update meetings with decision-makers helped build long-term relationships that proved valuable for the advocacy effort and created opportunities for ongoing dialogue and collaboration.

The success of this advocacy campaign would not have been possible without crucial support from influential decision-makers in the Majilis, who significantly contributed to achieving the ultimate goal—the approval of a vaping market ban. Among the most impactful supporters at the parliamentary level was Mrs. Nurgul Tau, an experienced teacher and educator whose background significantly strengthened advocacy efforts at every internal political discussion, and two members of parliament (Dr. Guldara Nurumova and Dr. Zarina Kamassova) who provided valuable pro-health expertise as physicians from the early stage of the campaign in 2021. The most senior politician, Mr. Edil Zhanbyrshin, head of the Ecology Committee, was a vocal advocate and political leader from the beginning and throughout Majilis stage, as well as in the Senate (upper chamber of the parliament). Their active participation and influential voices in parliament played a critical role in effectively communicating the urgency and importance of the vaping ban to senior decision-makers in the Majilis and Senat, and to the wider public.

Critically, the direct impact of the Minister of Health of Kazakhstan, who consistently advocated for a complete ban on vaping products at the governmental level from 2022, proved to be a game-changing factor in driving momentum and securing the final policy decision.

In addition, the Coalition and Majilis champions successfully engaged key political parties, “Respublika” and “Amanat,” thereby increasing the number of supportive members of parliament to 18, which significantly increased political pressure and impacted the discussion. Champions were engaged through a personal and hands-on approach: by meeting them in person, showing concrete data and the internal components of a vape device, and clearly explaining its harmful effects on health. However, not all ministries and political parties endorsed the campaign. A group of members of parliament openly supported the vaping industry and sought to intervene during discussions within the Majilis. This opposition primarily consisted of influential Majilis members linked to the business community, including some prominent billionaires. They accused the authors of the ban amendment of harming the business environment and questioned the evidence regarding vaping-related health risks.

Additionally, this lobbying group of parliamentarians sent a letter directly to the Prime Minister of Kazakhstan, asserting that banning vaping products could lead to increased crime rates and greater demand for illegal products, demonstrating alignment with industry interests. The Coalition actively countered these lobbying tactics in the Majilis by publicly exposing and addressing industry-driven arguments through targeted Facebook communications and used the same unmasking tactics at the Senate stage.

### 3.7. The Key Milestones of the Campaign

#### 3.7.1. Early Stages: October–December 2021

The advocacy campaign officially began on 27 October 2021, during a meeting of the National Health Committee of Nur Otan—the largest political party in Kazakhstan, later renamed Amanat—under the leadership of the “Smokefree Kazakhstan” Coalition, where the urgent need to ban vaping was announced due to its significant public health risk—vaping had become an epidemic among the Kazakhstani youth. The proposal received immediate support from the committee members, the MoH, and public health leaders and attracted the interest of key supporters in parliament, who became catalysts for internal discussions.

During this period, the Coalition carefully reviewed and analyzed the comprehensive data needed to prepare the RRA application to support the advocacy campaign, ensuring consistency and credibility in its messaging [28]. All available international and local evidence, including market data, health impact studies, and official statistics on vaping, was systematically organized and communicated according to RRA requirements [28]. This preparation allowed the Coalition to highlight vaping’s severe health risks, emphasizing dangers such as high nicotine content and its association with serious respiratory illnesses such as EVALI, as well as industry manipulation tactics.

On the basis of this robust evidence, the Coalition developed targeted advocacy messages emphasizing the urgency of banning vapes entirely and compiled the first draft of the RRA which had to be submitted to the Ministry of National Economy (MNE) on behalf of the Government of Kazakhstan for approval as per official requirement procedure. As a key step, the Coalition drafted specific legislative amendments proposing a complete vaping market ban through revisions to Article 110 of Kazakhstan’s Healthcare Law. This thorough and data-driven approach significantly strengthened the case when engaging decision-makers and the public.

#### 3.7.2. Development Phase: January–December 2022

During this period, the Coalition submitted the full RRA application to the government, proposing amendments that included criminal liabilities as part of the comprehensive vaping market ban. Throughout the advocacy campaign, several critical meetings were held with government officials and representatives from the MNE, alongside multiple public hearings as part of the RRA review process. These hearings were particularly important, as they offered a platform for mothers, public health leaders, and medical professionals to share compelling testimonies about the health risks and social impacts of vaping.

While the Coalition continued to advocate actively for incorporating criminal penalties into the vaping ban, the consistent engagement of key stakeholders through these public hearings significantly strengthened the overall campaign message and influenced policy discussions, underscoring widespread public support for total vaping prohibition.

At this stage, the involvement of key political figures, particularly the parliamentarians Zarina Kamassova and Nurgul Tau, along with the MoH, was pivotal in advancing the campaign. Later, the Coalition successfully gained the support of more than 12 members of parliament, who co-authored the legislative proposal, advocating for a complete vaping ban, including provisions for criminal liability.

However, in the summer of 2022, the Coalition faced a significant challenge when one of its strongest advocates, Dr. Zarina Kamassova, left parliament due to parliamentary elections and membership renewal. She had been a central figure in raising awareness and pushing the vaping ban agenda forward at the parliamentary level. Following her departure, Dr. Guldara Nurumova was appointed as the new head of the parliamentary working group and quickly became actively and enthusiastically involved in advancing campaign efforts. Despite this change, momentum continued as the Coalition maintained strong advocacy efforts and networking among members of parliament. Following the announcements, Dr. Giniyat, the Minister of Health, provided ongoing support which ensured advocacy efforts remained stable, vocal, and effective.

#### 3.7.3. Culminating Events: January–December 2023

The Coalition’s third phase of advocacy began in response to continued challenges posed by the vaping epidemic. At the center of this phase was the approval process of a revised RRA application to the MNE which was highly influenced by the vaping industry. The updated RRA emphasized the vaping industry’s role as the primary driver behind the epidemic, highlighting its aggressive marketing tactics aimed at children and adolescents. To gather feedback, the document was presented at an online public hearing involving NGOs, experts, and other stakeholders. However, this hearing proved highly challenging for Coalition members because of significant opposition from industry-aligned groups.

During the hearing, the Expert Council under the MNE allowed pro-vaping and tobacco industry front groups to actively intervene, prompting strong protests from Coalition members and the MoH. In response, 12 NGOs and two independent experts submitted formal protest letters to the Minister of National Economy, demanding a higher-level review of the RRA and fair offline discussion.

Following these protests, the Coalition secured a crucial in-person debate with pro-tobacco experts under moderation of the First Vice-Minister of the MNE, where they successfully advocated for including criminal liability measures against vape market operators as part of the comprehensive ban. After incorporating these critical amendments, the final RRA draft was submitted to the MNE in June 2023. Four-hour debates at which the Coalition effectively opposed every industry argument subsequently resulted in MNE approval of the RRA and the document being forwarded to the governmental committee for final review.

As the legislative process advanced, extensive deliberations were required to secure government support for the proposed ban. Out of a total of eleven formal consultations, three in-depth discussions—each lasting at least two hours—were held at the MNE to justify the inclusion of criminal liability in the ban. The MoH submitted six versions of the RRA to the MNE in an effort to overcome resistance and gain final approval. In parallel, three high-level meetings were convened with senior government officials to persuade key decision-makers of the need for strong regulatory measures. The Coalition and the MoH, working in tandem, played an extremely crucial role in defending every aspect of the RRA in public and political discussion.

Thus, in July 2023, the Government of Kazakhstan officially approved the first major legislative step toward banning vaping products, flavorings, and liquids including the introduction of criminal liabilities for industry participants [38]. This decision marked a significant victory, clearly demonstrating the impact of the joint, persistent Coalition and MoH advocacy efforts. However, later, the tobacco industry attempted to exclude provisions on criminal liability from the RRA through internal debates, reportedly influenced by the Deputy Prime Minister, who selectively interpreted an official position from the Ministry of Justice to support this position [38]. However, the clause was ultimately reinstated during the September 2022 government review, following the personal intervention of Prime Minister Alikhan Smailov. The Prime Minister’s direct engagement on restoring criminal liability as a ban enforcement tool underscored the effectiveness of the Coalition’s advocacy strategy and public messaging in elevating the ban to a top-level policy priority.

The next critical step was to secure approval from the Parliament of Kazakhstan. After Dr. Zarina Kamassova’s departure, the new champion among parliamentarians, Nurgul Tau, a dedicated educator, and Zhanbyrshin Edil, an influential advocate, actively advanced the Coalition’s agenda within parliament. They both played a crucial role in reinforcing the campaign’s central message and further support at the parliament level.

#### 3.7.4. Final Phase: January–April 2024

Finally, on 24 January 2024, the plenary session of the Majilis, the lower chamber of parliament, approved the Healthcare Law amendment, introducing a comprehensive ban on vaping products in Kazakhstan, including all flavored liquids, and establishing criminal and administrative liabilities for violations. Representatives from the vaping industry continued to oppose this decision by proposing alternative amendments focused solely on regulating the labeling of vaping products. Their main justification was the financial hardship the industry would face due to outstanding bank loans taken to launch vaping businesses. However, this argument failed to gain sufficient support and was ultimately rejected by parliamentarians.

Following the Majilis approval, the upper chamber of parliament—the Senate—endorsed the amended Healthcare Law and submitted it to the President of Kazakhstan. A group of Majilis members from the business community intervened, accusing the authors of the “ban amendment” of killing the business environment and citing the lack of evidence for the harm of vapes. This lobbyist group of MPs wrote a letter to the Prime Minister of the country stating that the ban of vaping within the country might lead to increased crime and demand for illegal products, therefore clearly supporting the interests of the vaping industry.

After 32 months of relentless joint MoH–Coalition advocacy efforts, together with key champions in the Majilis and NGOs, the President of Kazakhstan officially signed the amendments into law in April 2024 [38]. This legislation included a complete market (import, production, sale, export) ban on flavored vaping products and vaping liquids, along with clear provisions for criminal and administrative penalties, marking a significant milestone in Kazakhstan’s public health policy.

## 4. Discussion

This advocacy campaign’s success in Kazakhstan, i.e., the enactment of comprehensive piece of legislation banning vaping products, provides significant insights aligned with global public health advocacy literature, underscoring the effectiveness of comprehensive, multisectoral coalitions, robust data-driven messaging, coordinated advocacy, public engagement, and effective use of media platforms to shift public opinion and influence policy outcomes [39,40,41,42,43,44,45,46,47].

Over 32 months of persistent advocacy, the Smokefree Kazakhstan Coalition, in trusted partnership with the Ministry of Health, mobilized diverse societal stakeholders, including parents, healthcare professionals, religious leaders, NGOs, and key policymakers. The key activities of the campaign are outlined in the table below (see Table 1). Despite facing opposition from the vaping industry and political lobbyists, the Coalition effectively leveraged evidence-based messaging, grassroots advocacy, and strategic no-cost media outreach to achieve policy change. These findings align with global evidence indicating that multi-stakeholder coalitions and strong political alliances are critical to successful tobacco control policy [42,43]. The use of social media as a low-cost, high-impact tool also mirrors strategies seen in successful public health campaigns elsewhere [44].

The application of the Power Prism framework as a post-campaign analytical lens was instrumental in understanding and clearly communicating the strategies and outcomes of the Coalition [16]. Although the framework was not explicitly employed during implementation, its structured approach helped highlight how coalition-building, grassroots mobilization, media engagement, and direct decision-maker advocacy collectively contributed to achieving a complete vaping ban.

Reflecting on the campaign through this lens also revealed several areas for improvement. One key gap was the absence of systematic fundraising and financial planning. While the Ministry of Health provided basic support, the lack of diversified financial resources limited outreach efforts, particularly in broader media engagement. This challenge is consistent with findings from other low- and middle-income countries where advocacy campaigns struggle to sustain operations without dedicated funding streams [45].

The campaign also highlighted the need for deeper analysis of political dynamics and bureaucratic processes. Despite support from influential parliamentarians and the Minister of Health, delays caused by industry-aligned actors and procedural bottlenecks in the regulatory review process slowed implementation. These findings support prior studies emphasizing the importance of political mapping and stakeholder analysis in advocacy planning [46].

Finally, the lack of a guiding framework at the outset limited initial coordination and efficiency, with Coalition members experiencing confusion about sequencing and responsibilities. This reinforces the importance of structured advocacy models like the Power Prism, which offer a roadmap for planning, role allocation, and sustained action [16,39,40]. The literature on coalition effectiveness emphasizes the value of early role clarification and strategic frameworks in maximizing advocacy outcomes [47].

## 5. Conclusions

The experience of Kazakhstan serves as a valuable model for countries seeking to confront the public health threats associated with vaping. The campaign demonstrated the power of broad coalition-building, low-cost digital advocacy, and strategic political engagement in driving policy change. Key lessons include the necessity of clearly structured advocacy frameworks, systematic fundraising, and deep political analysis to ensure resilience against industry opposition.

As of 2025, the vaping ban is in effect, and the Coalition continues to play a vital role in monitoring its enforcement. In partnership with the Ministry of Internal Affairs, the Coalition actively tracks online sales of vape products, reports violations, and monitors media and market responses to the legislation. These ongoing efforts ensure that the policy not only exists on paper but is meaningfully implemented, serving as a foundation for long-term public health protection and a blueprint for future advocacy campaigns.

## Figures and Tables

**Table 1:** Chronology of key advocacy campaign activities.

Period	Key Advocacy Activities
**Phase 1: Initiation** *October*–*December 2021*	**Launch of Campaign and Message Framing** Advocacy campaign formally initiated at Nur Otan Health Committee (October 2021). Initiated data collection and development of evidence-based messaging including EVALI, toxic chemicals, and youth access. First draft of RRA prepared.
**Phase 2: Development** *January*–*December 2022*	**RRA Submission and Stakeholder Mobilization** RRA drafted and repeatedly revised for MoE approval. Public hearings with strong participation of parents, doctors, and NGOs. Key champions emerged in parliament.
**Phase 3: Culmination** *January*–*December 2023*	**Confronting Industry Influence and Gaining Governmental Approval** Finalized and resubmitted RRA with criminal liability provisions. Formal protests submitted by NGOs and experts. High-level negotiations with Vice-Minister and Prime Minister led to reinclusion of criminal liability. Legislative pathway in Parliament prepared with active support from MPs Nurgul Tau and Edil Zhambyrshin.
**Phase 4: Finalization** *January*–*April 2024*	**Parliamentary Approval and Presidential Endorsement** Majilis approved the Healthcare Law amendment including full vape ban and enforcement measures. Senate reviewed and strengthened enforcement language. Lobbying attempts from vape industry and business MPs countered through public exposure. April 2024: President signed amendment into law, marking full market ban with criminal and administrative liability.

## Data Availability

No new data were created.
